# Association between 24-hour movement guidelines and working memory in early Chinese adolescents

**DOI:** 10.3389/fpsyg.2025.1666581

**Published:** 2025-10-02

**Authors:** Peng Xue, Tong Han, Xinlan Jin, Wen Li, Yiyi Chen

**Affiliations:** ^1^Shandong Huayu University of Technology, Dezhou, China; ^2^Gdansk University of Physical Education and Sport, Pomeranian Voivodeship Gdańsk, Gdańsk, Poland; ^3^Taiyuan No.48 Middle School, Taiyuan, China; ^4^Jishou University, Jishou, China

**Keywords:** 24-hour movement guidelines, working memory, screen time, sleep, physical activity, early adolescence

## Abstract

**Objective:**

This study aimed to examine the association between adherence to the 24-hour movement behavior guidelines and WM performance in early adolescents, particularly the cumulative and gender-specific effects.

**Methods:**

A cross-sectional study was conducted on 2,163 adolescents aged 11–14 years. Participants’ adherence to the 24-hour movement guidelines was assessed using validated questionnaires. WM performance was measured via a computerized N-back task, including 1-back and 2-back conditions, with reaction time recorded as the primary outcome indicator. Statistical analyses (including descriptive statistics and regression analysis) were performed to explore the relationship between guideline adherence and WM performance, and gender-stratified analysis was further conducted.

**Results:**

Only 2.9% of participants met none of the 24-hour movement guidelines, while 27.2% met all three guide-lines; significant gender differences in adherence were observed (*p* < 0.05). A significant negative dose-response relationship was found between the number of guidelines met and reaction times in the N-back tasks (*p* < 0.01). The above dose-response effect was only significant in girls (2-back task: F = 15.095, *p* < 0.001), with no significant differences detected in boys. The dose-response trend was more pronounced under higher cognitive load conditions (2-back task, *p* < 0.01). Adolescents who met both screen time and sleep recommendations exhibited the best WM performance (shortest reaction time, *p* < 0.05).

**Conclusion:**

This study identifies a notable, dose-dependent association between adherence to multiple components of 24-hour movement behavior guidelines and better WM performance in early adolescence, with this pattern being particularly evident under high cognitive load conditions and girls showing greater sensitivity. The findings indicate that adherence to the 24-hour movement guidelines, particularly the combined reduction of screen time and adequate sleep duration, is associated with better WM performance during early adolescence.

## 1 Introduction

Working memory (WM) is a limited-capacity memory system responsible for the temporary storage and manipulation of information. It consists of four components: the central executive, phonological loop, visuospatial sketchpad, and episodic buffer (Baddeley, 2012). As a core component of executive functions, WM undergoes critical development during childhood and adolescence. It is not only considered an essential indicator of cognitive development (Diamond, 2020; Morra et al., 2025) but also acts as a key driver of this development, with significant influences on fluid intelligence (Ge et al., 2013) and academic performance (Forsberg et al., 2021) during early adolescence. Recent studies have highlighted the plasticity of WM, particularly during early adolescence (Lambek and Shevlin, 2011). Therefore, training and improving WM have become important targets for educational and clinical interventions, including mindfulness and computerized cognitive training (Tang et al., 2012; Jordan et al., 2020). However, these methods may only be applicable to specific populations, and their benefits may not generalize beyond the training tasks (Ludyga et al., 2022). For instance, in an 8-week intervention study conducted by Dong et al. (2023), mindfulness-based interventions were found to enhance WM solely in young adults with depression, while demonstrating no measurable effects on depressed adolescents. A meta-analysis of controlled studies indicates that mindfulness training yields minimal impact on WM and long-term memory in the general population (Im et al., 2021). Furthermore, empirical evidence suggests that the transfer effects of direct WM training through cognitive approaches remain contentious, with no definitive assurance regarding their efficacy in academic applications (Schwaighofer et al., 2015). Consequently, a cost-effective and convenient intervention suitable for a broader adolescent population is necessary to ensure more individuals can benefit from WM enhancements.

Over the past decade, lifestyle interventions have gained increasing attention as a means of promoting physical, psychological, social, and cognitive health among adolescents (Faulkner et al., 2016; Chaput et al., 2020; Sampasa-Kanyinga et al., 2020; Tapia-Serrano et al., 2023; Su et al., 2024). Within these lifestyle behaviors, 24-hour movement behaviors-encompassing physical activity (PA), sedentary behavior, and sleep-are critical factors influencing WM. A cross-sectional study has shown that, after adjusting for sociodemographic factors, age, gender, and body mass index (BMI), adolescents’ PA levels and sleep quality significantly impact their WM (Tee et al., 2018). Additionally, a prospective study reported that low extracurricular PA and high sedentary behavior at age six negatively correlated with WM at age 14 (López-Vicente et al., 2017). Moreover, Kawaike et al. (2019) demonstrated using wearable functional near-infrared spectroscopy devices that hemodynamic activity in the prefrontal cortex during WM tasks could be modulated by daily internet usage and weekly PA frequency. These studies indicate individuals with higher levels of PA, lower sedentary behavior, and adequate sleep exhibit greater brain activation and better WM performance during cognitive processing.

While the impact of individual components within 24-hour movement behaviors on WM has been validated across populations such as children and adolescents, a growing number of scholars have advocated for a unified framework that conceptualizes PA, sedentary behavior, and sleep as interconnected “24-hour movement behaviors”-a holistic construct emphasizing their dynamic interactions within daily cycles (Rosenberger et al., 2019). This perspective emerged from observations that these behaviors do not operate in isolation: for example, Ramer et al. (2022) found sleep significantly moderates the relationship between early PA and later cognition, weakening the association at higher sleep levels. Such interdependencies make it challenging to disentangle their individual effects (Chastin et al., 2015; Chaput et al., 2017). In line with this integrated framework, the 24-hour movement guidelines were developed to synthesize recommendations for PA, screen time, and sleep into a cohesive approach, moving beyond siloed advice on single behaviors (Tremblay et al., 2016; Tapia-Serrano et al., 2022). Specifically, for children and adolescents aged 5–17 years (the age range targeted by the guidelines), these recommendations include: (1) engaging in at least 60 min of MVPA daily; (2) limiting recreational screen time to less than 2 h per day (it is important to note that the screen time specified here refers explicitly to recreational use, excluding screen time for academic or work purposes); and (3) obtaining age-specific sleep duration 9–11 h per night for children aged 5–13 years, and 8–10 h per night for adolescents aged 14–17 years.

However, research exploring the combined effects of these three behaviors on WM remains limited. Most existing studies have focused on general cognitive outcomes from a holistic “24-hour movement behavior” perspective, with few studies specifically targeting the integrated benefits of these behaviors on particular cognitive domains in early adolescence. For example, Walsh et al. (2018) assessed associations between adherence to 24-hour movement guidelines and general cognitive performance, demonstrating better overall cognitive outcomes in children meeting all three guidelines, screen-time only, or both screen-time and sleep recommendations, compared to those meeting none. Similarly, a combined cross-sectional and longitudinal study found that, in the cross-sectional analysis, early adolescents adhering fully to the guidelines-particularly those meeting both sleep and screen-time recommendations-exhibited better cognitive scores and greater grey matter volumes compared to those meeting none of the recommendations. The longitudinal follow-up (with a 2-year interval between T1 and T2) further supported these findings, as participants who met both recommendations at both time points showed superior outcomes relative to those who met none across the study period (Fung et al., 2023).

Collectively, these findings emphasize the importance of adopting an integrated 24-hour movement perspective for promoting cognitive health in early adolescence, rather than focusing solely on individual behavior recommendations. Therefore, the present study investigates the associations between 24-hour movement behaviors and WM from a holistic perspective, aiming to elucidate the comprehensive impact of healthy lifestyles on WM health and provide scientific evidence for developing more integrated intervention strategies.

## 2 Materials and methods

### 2.1 Research design and participants

This study is based on the baseline survey of a school-based prospective cohort study conducted in Taiyuan, China, during April–May 2025. Firstly, schools were categorized into three levels (high, medium, and low) according to the evaluation results of compulsory education quality in Taiyuan. This classification was incorporated to provide contextual information about the educational setting and to increase transparency regarding the sampling framework, ensuring that the selected schools were representative of the diverse educational settings within Taiyuan’s compulsory education system. From each level, 3 middle schools were randomly selected, resulting in a total of 9 schools for sampling. Subsequently, in each selected school, classes from Grade 7 and Grade 8 were further chosen using random sampling, with 3 to 4 classes selected per grade, totaling 68 classes. All students in these selected classes were included in the study. After excluding invalid and missing samples, 2,163 participants were included in the statistical analysis. The study cohort comprised 1,131 male and 1,032 female participants, with an age range spanning from 11 to 14 years. Students participating in the study and their guardians have signed informed consent forms. This study conforms to the Declaration of Helsinki and has been approved by the Biomedical Ethics Committee of Jishou University (Approval No.: JSDX-2025-0063).

### 2.2 Procedure

Data collection was conducted by trained members of the research team, with coordination from class teachers to ensure consistency across participants. Questionnaire assessments (including measures of 24-hour movement behaviors, demographic information, and covariates) were administered collectively during regular class meetings. Research staff distributed paper questionnaires to students, provided standardized verbal instructions to clarify item interpretations, and remained present to address questions. Completed questionnaires were collected immediately after completion to ensure full retrieval. For the evaluation of WM, assessments via the online N-back task were conducted individually during scheduled computer class periods. Each student completed the task on a separate computer in the school’s computer laboratory, with research staff monitoring to ensure adherence to task instructions and minimize distractions. Prior to formal testing, participants completed practice task to familiarize themselves with the task protocol, and testing sessions lasted approximately 10–15 min per student.

### 2.3 Measures

#### 2.3.1 24-hour movement behavior

The PA and screen time of adolescents were assessed using the health industry standard “Physical Activity Level Evaluation for Children and Adolescents Aged 7–18 Years,” issued by the Chinese National Bureau of Disease Prevention and Control (National Health Commission of the People’s Republic of China (National Center for Disease Control and Prevention), 2024).

The PA assessment included 24 items across various activities, such as ball games, aerobics, outdoor games, swimming, cycling, running, and household chores. Information on activity intensity, duration, and frequency was collected via self-reported recall by adolescents, who documented their engagement in these activities over the past week. Activity intensity, duration, and frequency were evaluated. Total activity duration was calculated by multiplying the frequency of each activity by its average duration per session. Intensities of PA were categorized based on metabolic equivalents of energy (METs), defined as follows: light-intensity physical activity between 1.5 and 3 METs, moderate-intensity physical activity (MPA) between 3 and 6 METs, and vigorous-intensity physical activity (VPA) exceeding 6 METs (Ainsworth et al., 2011). The validity of this questionnaire was verified in previous study (Wu et al., 2023). The results indicated that the reliability of the items for MVPA and total physical activity (TPA) was relatively high: the Cronbach’s *α* coefficients were 0.70 and 0.72, respectively, (both *p* < 0.01), and the test–retest reliability coefficients were 0.74 for MVPA and 0.78 for TPA (both *p* < 0.01). The correlation coefficient between the questionnaire and the MVPA measured by the accelerometer was 0.69 (*p* < 0.01), which demonstrated the good validity of the questionnaire. Levels of MVPA were categorized as meeting (≥ 60 min/day) or not meeting (< 60 min/day) the recommended guidelines (Guthold et al., 2020).

Screen time was assessed through self-reported questions, which covered the frequency and duration of recreational television viewing, as well as the recreational use of computers, smartphones, or tablets. Screen time used for educational purposes mandated by educational institutions-specifically, online video teaching arranged by educational authorities via television, computers, or mobile devices/tablets-was excluded, in accordance with the criteria outlined in the PA level evaluation for children and adolescents aged 7–18 years (National Health Commission of the People’s Republic of China (National Center for Disease Control and Prevention), 2024). Participants with more than 2 h of screen time per day were classified as not meeting the screen time recommendation, while those with 2 h or less per day were classified as meeting the recommendation (van Sluijs et al., 2021).

Sleep duration was measured based on the Pittsburgh Sleep Quality Index (PSQI), including bedtime, wake-up time, and sleep latency during weekdays and weekends. Consistent with the PSQI scoring protocol, sleep latency was operationalized as the categorical self-report of the time taken to fall asleep, with response options including “≤15 min,” “16–30 min,” “31–60 min,” and “> 60 min.” These categorical responses were converted to numerical values (e.g., “≤15 min” = 0.25 h, “16–30 min” = 0.5 h, “31–60 min” = 0.75 h, “> 60 min” = 1.25 h) for quantitative calculation. Sleep duration was calculated as follows: sleep duration (hours) = (wake-up time – bedtime) – sleep latency. Participants reported separate sleep parameters for a typical weekday and a typical weekend day. Weekly average sleep duration was then calculated using a weighted formula to account for the 5:2 distribution of weekdays and weekends in a week. Weekly sleep duration was determined by: [5 × (weekday sleep duration) + 2 × (weekend sleep duration)] / 7. According to the regulations of the Chinese Ministry of Education and related research results, adolescents who met the recommendations for sleep (8–10 h per night) were classified as achieving adequate sleep in this study; otherwise, they were classified as not meeting the recommendations (Ge et al., 2025).

Participants were categorized as 0 = meeting none of movement behaviors, 1 = meeting one recommendation of movement behaviors, 2 = meeting two recommendations of movement behaviors, and 3 = meeting all three recommendations of movement behaviors.

#### 2.3.2 Working memory

The N-back task paradigm was used to evaluate the performance in WM. The 1-back test involves the sequential presentation of five letters (A, S, P, G, T) in random order at the center of the screen. Participants are required to promptly press the “F” key when the currently displayed letter matches the immediately preceding one, and the “J” key when no match is detected. Each stimulus remains visible for 2,000 ms, with an inter-stimulus interval of 3 s. In the 2-back test paradigm, participants must respond to matches occurring with one intervening letter, pressing “F” for matches and “J” for non-matches. The temporal parameters and testing requirements remain identical to those of the 1-back protocol. Each testing phase comprises both practice trials and formal assessments, with the latter consisting of 25 stimulus presentations. Reaction times for correct responses were recorded as the primary outcome, with shorter reaction times indicating superior WM performance. The N-back tasks were administered using the computerized executive function testing system (APACHE, version 2.2) developed by Yangzhou University (Zhu et al., 2020).

#### 2.3.3 Covariates

Covariates included general demographic information and family environmental factors. Demographic data encompassed adolescents’ birth date, gender, grade, and education stage. Height and weight were obtained from school health examination records and used to calculate BMI. The health examination was conducted from March to April in 2025. Family environment assessments included family structure, parental education levels, parental occupations, and household facilities. Socioeconomic status (SES) was derived from parental education, occupation, and household facilities, classified into three categories (low, medium, high) based on tertile distributions (Ding et al., 2023).

### 2.4 Statistical analysis

Data management and statistical analysis were performed using SPSS software (version 26.0; IBM Corp., Armonk, NY, USA). Descriptive analyses were conducted for categorical data, presented as frequencies and percentages (%), and continuous data, presented as means ± standard deviations. Multiple linear regression models were employed to examine the associations between adherence to 24-hour movement guidelines and WM performance. The dependent variables were WM reaction times on the 1-back and 2-back tasks, analyzed separately to account for differences in cognitive load; the independent variables were operationalized as two indicators of guideline adherence: (1) the total number of guideline components met (0, 1, 2, or 3) and (2) specific combinations of met components (e.g., meeting PA and sleep guidelines but not screen time guidelines); covariates included as control variables were: age, sex, grade, educational stage, BMI, family environment factors and SES. For analyses investigating the total number of met guideline components, sex-stratified regression models were applied to explore potential gender-specific patterns, consistent with observations from descriptive analyses. Statistical significance was set at *p* < 0.05.

## 3 Results

### 3.1 Basic characteristics of the samples

The basic characteristics of the samples are presented in Table 1. The prevalence of achieving MVPA was 50.49%, significantly higher among boys (54.91%) compared to girls (45.64%) (*p* < 0.001). The prevalence of meeting the recommended screen time was 81.83%, slightly lower in boys (80.46%) compared to girls (83.33%), but the difference was not statistically significant (*p* = 0.083). The proportion meeting the recommended sleep duration was 65.37%, slightly higher among boys (67.02%) than girls (63.57%), with no significant gender difference (*p* = 0.092). Only 2.87% of adolescents met none of the 24-hour movement guidelines (boys 2.21%, girls 3.59%), whereas 27.18% met all guidelines (boys 29.9%, girls 24.13%). Significant gender differences were observed in the distribution of meeting the number of guidelines (*p* = 0.008).

**Table 1:** Descriptive characteristics of the participants.

Variables	Total^a^	Boys^a^	Girls^a^	*P* ^b^
	(*N* = 2,163)	(*N* = 1,131)	(*N* = 1,032)	
Age (years)	13.86 ± 0.72	13.89 ± 0.74	13.82 ± 0.70	0.021
BMI	20.70 ± 4.37	21.12 ± 4.61	20.23 ± 4.04	<0.001
Family structure				0.362
Core family	73.19%(1,583)	74.54%(843)	71.71%(740)	
Extended family	21.22%(459)	20.60%(233)	21.90%(226)	
Joint family	0.69%(15)	0.53%(6)	0.87%(9)	
Single-parent family	2.87%(62)	2.74%(31)	3.00%(31)	
Reconstituted family	2.03%(44)	1.59%(18)	2.52(26)	
Family socioeconomic status				0.504
Low	40.64%(879)	41.56%(470)	39.63%(409)	
Middle	25.01%(541)	25.20%(285)	24.81%(256)	
High	34.35%(743)	33.24(376)	35.56%(367)	
PA				<0.001
Meet	50.49%(1,092)	54.91%(621)	45.64%(471)	
Do not meet	49.51%(1,071)	45.09%(510)	54.36%(561)	
Screen time				0.083
Meet	81.83%(1770)	80.46%(910)	83.33%(860)	
Do not meet	18.17(393)	19.54%(221)	16.67%(172)	
Sleep duration				0.092
Meet	65.37%(1,414)	67.02%(758)	63.57%(656)	
Do not meet	34.63%(749)	32.98%(373)	36.43%(376)	
Number of guidelines met				0.008
Three	27.18(588)	29.97%(339)	24.13%(249)	
Two out of three	46.19(999)	44.65%(505)	47.87%(494)	
One out of three	23.76%(514)	23.17%(262)	24.42%(252)	
None	2.87%(62)	2.21%(25)	3.59%(37)	

a The data shown in percentage for categorical variables and mean (SD) for continuous variables.

b *p* value was calculated based on Chi-squared test for categorical variables and t-test for continuous variables.

BMI, Body Mass Index; PA, Physical Activity; P, Probability.

### 3.2 Working memory characteristics of different numbers of items meeting the standards

The WM characteristics of samples with varying numbers of met criteria are presented in Table 2. Adolescents who met all three 24-hour movement guidelines had reaction times of 800.448 ± 327.010 ms and 991.879 ± 399.898 ms for 1-back and 2-back tasks, respectively. In contrast, adolescents meeting none of the guidelines had reaction times of 921.329 ± 285.270 ms (1-back) and 1148.444 ± 382.324 ms (2-back). A significant linear relationship between the number of guidelines met and WM performance was observed (1-back: *F* = 10.062, *p* = 0.002; 2-back: *F* = 11.408, *p* = 0.001). When stratified by gender, this linear relationship was not significant among boys (1-back: *F* = 1.648, *p* = 0.200; 2-back: *F* = 0.157, *p* = 0.692) but was significant among girls (1-back: *F* = 8.472, *p* = 0.004; 2-back: *F* = 15.095, *p* < 0.001).

**Table 2:** Comparison of working memory performance according to the number of 24-hour movement guidelines met.

Variables	None (*n* = 62)	One out of three (*n* = 514)	Two out of three (*n* = 999)	Three (*n* = 588)	*F*	*P* for trend
Total						
1-back	921.329 ± 285.270	866.579 ± 1289.591	832.064 ± 310.730	800.448 ± 327.010	10.062	0.002
2-back	1148.444 ± 382.324	1089.294 ± 338.976	1041.712 ± 385.291	991.879 ± 399.898	11.408	0.001
Boys						
1-back	860.783 ± 350.329	869.764 ± 291.271	819.898 ± 298.765	793.442 ± 346.354	1.648	0.200
2-back	994.018 ± 481.415	1055.642 ± 344.353	1026.264 ± 382.355	972.020 ± 409.320	0.157	0.692
Girls						
1-back	962.238 ± 227.610	863.266 ± 288.377	844.502 ± 322.332	809.392 ± 305.990	8.472	0.004
2-back	1252.787 ± 255.777	1124.282 ± 330.351	1057.504 ± 388.020	1018.915 ± 385.874	15.095	0.000

F, Fisher; P, Probability.

### 3.3 The relationship between 24-hour activity guidelines and working memory

The results of the multiple linear regression analysis between 24-hour activity guidelines and WM are presented in Table 3. Adolescents meeting all three 24-hour movement guidelines had significantly shorter reaction times compared to those meeting fewer or no guidelines. For 1-back tasks, adolescents meeting one guideline had an *B* of 66.131 (95% CI: 29.454–102.808), and those meeting none had an *B* of 120.881 (95% CI: 39.776–201.985). For 2-back tasks, *B* was 49.833 (95% CI: 11.216–88.451) for those meeting two guidelines, 97.416 (95% CI: 52.553–142.278) for one guideline, and 156.566 (95% CI: 57.360–255.772) for no guidelines (all *p* < 0.05). Moreover, a statistically significant interaction effect was observed between sex and the number of guidelines met in the 2-back task (*B* = 18.441, 95% CI: 3.353–33.530; *p* < 0.05).

**Table 3:** Associations between meeting the PA, screen time, and sleep duration recommendations and working memory.

Guidelines	1-back		2-back	
	*B*(95% CI)	*P*	*B*(95% CI)	*P*
Number of guidelines met				
Three	Reference		Reference	
Two out of three	31.616(0.045, 63.187)	0.050	49.833(11.216, 88.451)	0.011
One out of three	66.131(29.454, 102.808)	0.000	97.416(52.553, 142.278)	0.000
None	120.881(39.776, 201.985)	0.004	156.566(57.360, 255.772)	0.002
Sex × Number of Guidelines Met	7.679(−4.668, 20.027)	0.223	18.441(3.353, 33.530)	0.017
PA				
Meet	Reference		Reference	
Do not meet	−0.072(−28.287, 24.143)	0.877	18.893(−13.205, 50.990)	0.249
ST				
Meet	Reference		Reference	
Do not meet	116.511(82.876, 150.146)	0.000	78.164(36.662, 119.665)	0.000
Sleep duration				
Meet	Reference		Reference	
Do not meet	22.347(−5.185, 49.879)	0.112	64.539(30.909, 98.169)	0.000
PA + ST				
Meet	Reference		Reference	
Do not meet	33.819(7.122,60.516)	0.013	37.564(4.858, 70.270)	0.024
PA + Sleep duration				
Meet	Reference		Reference	
Do not meet	9.118(−18.617, 36.853)	0.519	42.913(8.989, 76.837)	0.013
ST + Sleep duration				
Meet	Reference		Reference	
Do not meet	73.997(47.888, 100.105)	0.000	92.876(60.910, 124.840)	0.000
All three recommendations				
Meet	Reference		Reference	
Do not meet	46.393(16.999, 75.786)	0.002	69.563(33.601, 105.252)	0.000

PA, Physical Activity; ST, Screen Time; P, Probability; B, Unstandardized Coefficient B; CI, Confidence Interval.

Adolescents not meeting the recommended screen time had significantly longer WM reaction times (1-back: *B* = 116.511, 95% CI: 82.876–150.146; 2-back: *B* = 78.164, 95% CI: 36.662–119.665; *p* < 0.05). Additionally, those not meeting sleep duration recommendations showed significantly longer reaction times in the 2-back task (*B* = 64.539, 95% CI: 30.909–98.169; *p* < 0.05).

Compared to adolescents meeting both PA and screen time recommendations, those not meeting these criteria had longer reaction times (1-back: *B* = 33.819, 95% CI: 7.122–60.516; 2-back: *B* = 37.564, 95% CI: 4.858–70.270; *p* < 0.05). Similarly, not meeting both PA and sleep duration recommendations resulted in significantly longer 2-back reaction times (*B* = 42.913, 95% CI: 8.989–76.837; *p* < 0.05). Adolescents not meeting both screen time and sleep duration recommendations exhibited longer reaction times (1-back: *B* = 73.997, 95% CI: 47.888–100.105; 2-back: *B* = 92.876, 95% CI: 60.910–124.840; *p* < 0.05). Lastly, adolescents who failed to meet all three guidelines had significantly longer reaction times compared to those who met all (1-back: *B* = 46.393, 95% CI: 16.999–75.786; 2-back: *B* = 69.563, 95% CI: 33.601–105.252; *p* < 0.05).

## 4 Discussion

This study examined the relationship between adherence to 24-hour movement guidelines and WM performance among early adolescents. We identified a significant dose–response relationship between the number of guidelines met and WM, with notable gender differences. Furthermore, this association was particularly evident under high cognitive load conditions. Different combinations of meeting guidelines resulted in varying WM performances, with the combination of meeting screen time and sleep duration recommendations being optimal. These findings suggest that adherence to comprehensive 24-hour movement guidelines is associated with better cognitive outcomes, including WM, in early adolescents. Such observations highlight the potential value of exploring movement behavior promotion as a promising strategy to support cognitive development in this population, though further experimental research is needed to confirm causal relationships and evaluate intervention effectiveness.

The observed association suggests a potential linear pattern, with higher adherence to guideline-recommended behaviors being linked to better WM performance. This points to possible cumulative cognitive benefits associated with adequate PA, sufficient sleep, and moderate screen time, reflecting the integrated nature of healthy lifestyle behaviors. Such associations might be explained by plausible mechanisms, including increased neural plasticity and enhanced attentional resources that could be promoted by regular exercise and sufficient sleep-though these remain theoretical at this stage (Pickersgill et al., 2022; Amtul and Atta-Ur-Rahman., 2015). These findings are consistent with existing literature; for example, a systematic review noted that adherence to both screen time and sleep guidelines was associated with superior fluid intelligence and increased gray matter volume compared to adherence to single behaviors. Additionally, meeting all guidelines showed a consistent positive correlation with cognitive outcomes, which further supports the possibility of a linear relationship between healthy behaviors and cognition (Liu et al., 2025).

Interestingly, while a significant dose–response trend emerged in the overall sample, this relationship reached statistical significance only among girls-a finding that points to potential gender-specific underlying mechanisms. Neuroscience studies indicate gender differences in brain networks activated during WM tasks, even with comparable performance (Zayed and Jansen, 2018). Hormonal differences and variations in brain development trajectories during adolescence-such as earlier peak brain volume in girls and structural regional differences-may contribute to gender-specific cognitive responses to lifestyle behaviors (Lenroot and Giedd, 2010). At the behavioral level, gender differences in lifestyle patterns among adolescents are multifaceted. For instance, males typically engage in more screen time and PA (Guthold et al., 2020; van Sluijs et al., 2021). With respect to sleep, gender-related variations are complex and inconsistent across studies (Hossian et al., 2025), with some reporting longer sleep duration in males during weekdays and in females during weekends (Sanz-Martín et al., 2022). These findings collectively highlight the complexity of gender-specific lifestyle configurations rather than simplistic quantitative differences in individual behaviors. Such nuanced lifestyle distinctions may underpin the observed gender specificity in the association between behavior and cognition.

The stronger relationship observed under high cognitive load suggests that complex cognitive tasks are more sensitive to individual differences in cognitive resource utilization. In simple tasks, participants may approach maximal performance (ceiling effect), limiting group distinctions. In contrast, high cognitive load tasks demand substantial executive control and attentional resources-domains that overlap with those potentially influenced by PA and sleep (Sewell et al., 2021). Our findings align with research linking physical activity-related behaviors to enhanced performance in tasks requiring executive control, a broader domain that includes both inhibitory processes and WM. For example, study has noted that adolescents engaging in more PA tend to exhibit better performance in tasks demanding executive regulation, such as managing attentional focus and coordinating cognitive demands (Herting and Chu, 2017). While Huang et al. (2015) specifically reported shorter reaction times in adolescents with higher cardiorespiratory fitness during interference inhibition tasks (a component of executive control), their findings resonate with ours in highlighting that activity-related behaviors may support cognitive efficiency under high demands. Regarding sleep, research indicates that adolescents also showed impaired performance and diminished brain responses during the hardest task level (3-back) under a week of chronic sleep restriction (Alsameen et al., 2021). This provides a plausible mechanism for why groups with better adherence to sleep guidelines (i.e., sufficient sleep duration) demonstrated greater cognitive advantages in high-load conditions, as adequate sleep may support the executive resources needed for complex tasks.

However, distinct behavioral combinations can modulate the impact of health behaviors on health outcomes, with such moderation effects exhibiting variations across different outcome categories. A systematic review revealed that when assessing PA and sedentary behavior alone, the optimal combination comprises high PA coupled with low sedentary levels. Nevertheless, when sleep duration is incorporated into the analysis, behavioral patterns including adequate sleep typically demonstrate superior outcomes (Wilhite et al., 2023). For instance, Guerrero et al. (2019) demonstrated that children adhering to both screen time and sleep guidelines exhibited reduced impulsive behaviors across multiple dimensions (e.g., urgency, perseverance, behavioral inhibition system). Furthermore, evidence from a systematic review indicates compliance with screen time and sleep recommendations correlates with enhanced fluid intelligence and greater gray matter volume (Liu et al., 2025). Our findings similarly suggest that the “screen time + sleep duration” composite in adolescents predicts better WM performance. The underlying mechanisms involve two complementary pathways: excessive screen exposure has been empirically shown to impair executive functions, particularly WM capacity, with studies documenting how media multitasking degrades adolescents’ WM and inhibitory control (Muppalla et al., 2023). Conversely, chronic sleep deprivation is well-established to disrupt WM functionality, reducing both capacity and processing efficiency (Alsameen et al., 2021). Consequently, adherence to screen time and sleep guidelines may mitigate these detrimental effects, thereby enhancing cognitive performance.

Regarding PA, we observed limited independent cognitive benefits, consistent with recent reviews suggesting uncertainty around the direct relationship between PA guideline adherence and cognitive outcomes (Erickson et al., 2019). This suggests that potential benefits of PA in our study may have been overshadowed by screen time or sleep behaviors. Indeed, associations between PA characteristics-such as duration, intensity, and type-and cognitive outcomes are complex (Shi et al., 2022). For example, while MPA (e.g., 30–60 min of brisk walking) has been linked to improved WM in adolescents, excessively long durations (e.g., >90 min of continuous exercise) may not yield proportional benefits and could even be associated with transient cognitive fatigue due to energy depletion (Diamond and Ling, 2016). In terms of intensity, high-intensity interval training shows promise for enhancing executive function in short bouts, but its effects appear to differ from those of moderate-intensity steady-state exercise, which may more consistently support long-term cognitive resilience (Pesce et al., 2016). These nuances highlight that the cognitive impact of PA is not uniform but depends on a interplay of quantitative and qualitative factors. Nonetheless, despite limited independent effects, promoting adequate PA remains valuable. Schools might consider integrating short-duration, high-intensity PA breaks (e.g., 3–5 min of skipping rope, brisk walking, or aerobic games) into lessons to interrupt prolonged sedentary behavior, as such frequent and brief PA interruptions have been shown to enhance WM in adolescents (Feter et al., 2024). For example, regular short aerobic exercises during class have been found to improve performance on cognitively demanding tasks compared to prolonged sitting (Kjellenberg et al., 2024). Thus, classroom exercises or short runs during breaks can help maintain moderate heart rate elevations, optimizing cerebral blood flow and cognitive function. Overall, these findings suggest that multidisciplinary intervention strategies integrating PA, reduced screen time, and adequate sleep may hold promise for supporting cognitive development among early adolescents, with further research needed to quantify their combined effects on cognitive outcomes.

### 4.1 Limitations

First, the cross-sectional design of this study precludes the establishment of causal relationships, as it only allows for the identification of associations between behaviors and WM. Reverse causality or unmeasured confounding may exist; for example, adolescents with stronger cognitive abilities may be more likely to engage in PA or regulate screen time (Liu et al., 2025). Second, data on PA, sleep duration, and screen time were primarily collected via self-reported questionnaires, which are susceptible to recall bias and potential inaccuracies in time estimation. Future studies should incorporate objective measurements (e.g., accelerometers, sleep monitors) to complement these assessments (Troiano et al., 2008; Fabbri et al., 2021). Third, WM was evaluated solely using reaction times from the N-back task, without consideration of accuracy rates or other cognitive domains. This may limit the comprehensiveness of our assessment of individual cognitive abilities. Fourth, the sample was restricted to urban Chinese adolescents within a specific age range, and the generalizability of our findings needs to be validated in populations with diverse cultural backgrounds and across different age groups. Additionally, potential confounding variables such as nutritional status and mental health were not controlled for in the current study. These factors should be incorporated into future research designs to enhance the robustness of the findings.

## Data Availability

The datasets presented in this article are not readily available because the data are part of an ongoing study. Requests to access the datasets should be directed to corresponding author.

## References

[ref1] Ainsworth B. E. Haskell W. L. Herrmann S. D. Meckes N. Bassett D. R. Tudor-Locke C.. (2011). 2011 compendium of physical activities: a second update of codes and MET values. Med. Sci. Sports Exerc. 43, 1575–1581. doi: 10.1249/MSS.0b013e31821ece12, PMID: 21681120

[ref2] Alsameen M. DiFrancesco M. W. Drummond S. P. A. Franzen P. L. Beebe D. W. (2021). Neuronal activation and performance changes in working memory induced by chronic sleep restriction in adolescents. J. Sleep Res. 30:e13304. doi: 10.1111/jsr.13304 33615598 PMC8462989

[ref3] Amtul Z. Atta-Ur-Rahman. (2015). Neural plasticity and memory: molecular mechanism. Rev. Neurosci. 26, 253–268. doi: 10.1515/revneuro-2014-0075 25995328

[ref4] Baddeley A. (2012). Working memory: theories, models, and controversies. Annu. Rev. Psychol. 63, 1–29. doi: 10.1146/annurev-psych-120710-100422, PMID: 21961947

[ref5] Chaput J. P. Saunders T. J. Carson V. (2017). Interactions between sleep, movement and other non-movement behaviours in the pathogenesis of childhood obesity. Obes. Rev. 18 Suppl 1, 7–14. doi: 10.1111/obr.12508, PMID: 28164448

[ref6] Chaput J. P. Willumsen J. Bull F. Chou R. Ekelund U. Firth J.. (2020). 2020 WHO guidelines on physical activity and sedentary behaviour for children and adolescents aged 5-17 years: summary of the evidence. Int. J. Behav. Nutr. Phys. Act. 17:141. doi: 10.1186/s12966-020-01037-z, PMID: 33239009 PMC7691077

[ref7] Chastin S. F. Palarea-Albaladejo J. Dontje M. L. Skelton D. A. (2015). Combined effects of time spent in physical activity, sedentary behaviors and sleep on obesity and cardio-metabolic health markers: a novel compositional data analysis approach. PLoS One 10:e0139984. doi: 10.1371/journal.pone.0139984, PMID: 26461112 PMC4604082

[ref8] Diamond A. (2020). Executive functions. Handb. Clin. Neurol. 173, 225–240. doi: 10.1016/B978-0-444-64150-2.00020-4 32958176

[ref9] Diamond A. Ling D. S. (2016). Conclusions about interventions, programs, and approaches for improving executive functions that appear justified and those that, despite much hype, do not. Dev. Cogn. Neurosci. 18, 34–48. doi: 10.1016/j.dcn.2015.11.005, PMID: 26749076 PMC5108631

[ref10] Ding J. Wan Q. Gan M. L. Yin X. J. Wu H. P. Ma Y. Y.. (2023). Association between family environment and depressive symptoms in adolescents. Chin. J. Sch. Health. 44, 677–681. doi: 10.16835/j.cnki.1000-9817.2023.05.009

[ref11] Dong M. Li Y. Zhang Y. (2023). The effect of mindfulness training on executive function in youth with depression. Acta Psychol. 235:103888. doi: 10.1016/j.actpsy.2023.103888, PMID: 36934696

[ref12] Erickson K. I. Hillman C. Stillman C. M. Ballard R. M. Bloodgood B. Conroy D. E.. (2019). Physical activity, cognition, and brain outcomes: a review of the 2018 physical activity guidelines. Med. Sci. Sports Exerc. 51, 1242–1251. doi: 10.1249/MSS.0000000000001936, PMID: 31095081 PMC6527141

[ref13] Fabbri M. Beracci A. Martoni M. Meneo D. Tonetti L. Natale V. (2021). Measuring subjective sleep quality: a review. Int. J. Environ. Res. Public Health 18:1082. doi: 10.3390/ijerph18031082, PMID: 33530453 PMC7908437

[ref14] Faulkner G. White L. Riazi N. Latimer-Cheung A. E. Tremblay M. S. (2016). Canadian 24-hour movement guidelines for children and youth: exploring the perceptions of stakeholders regarding their acceptability, barriers to uptake, and dissemination. Appl. Physiol. Nutr. Metab. 41, S303–S310. doi: 10.1139/apnm-2016-0100 27306436

[ref15] Feter N. Ligeza T. S. Bashir N. Shanmugam R. J. Montero Herrera B. Aldabbagh T.. (2024). Effects of reducing sedentary behaviour by increasing physical activity, on cognitive function, brain function and structure across the lifespan: a systematic review and meta-analysis. Br. J. Sports Med. 58, 1295–1306. doi: 10.1136/bjsports-2024-108444, PMID: 39197948

[ref16] Forsberg A. Adams E. Cowan N. (2021). “The role of working memory in long-term learning: implications for childhood development” in The psychology of learning and motivation, vol. 74, 1–45.

[ref17] Fung H. Yeo B. T. T. Chen C. Lo J. C. Chee M. W. L. Ong J. L. (2023). Adherence to 24-hour movement recommendations and health indicators in early adolescence: cross-sectional and longitudinal associations in the adolescent brain cognitive development study. J. Adolesc. Health 72, 460–470. doi: 10.1016/j.jadohealth.2022.10.019, PMID: 36528521

[ref18] Ge Z. L. Dang J. X. Li J. Gao X. C. Zhang F. C. (2013). Study on the relationship of working memory, central executive function and general fluid intelligence. J. Zhejiang Univ. Sci. Ed. 40, 102–105. doi: 10.3785/j.issn.1008-9497.2013.01.021

[ref19] Ge D. Yao J. Zhou S. (2025). Effects of sleep duration on academic performance and its heterogeneity: evidence from 165,750 primary and secondary school students in China. Psychol. Sch. 62, 2542–2553. doi: 10.1002/pits.23484

[ref20] Guerrero M. D. Barnes J. D. Walsh J. J. Chaput J. P. Tremblay M. S. Goldfield G. S. (2019). 24-hour movement behaviors and impulsivity. Pediatrics 144:e20190187. doi: 10.1542/peds.2019-0187, PMID: 31413180 PMC6856835

[ref21] Guthold R. Stevens G. A. Riley L. M. Bull F. C. (2020). Global trends in insufficient physical activity among adolescents: a pooled analysis of 298 population-based surveys with 1·6 million participants. Lancet Child Adolesc. Health. 4, 23–35. doi: 10.1016/S2352-4642(19)30323-2, PMID: 31761562 PMC6919336

[ref22] Herting M. M. Chu X. (2017). Exercise, cognition, and the adolescent brain. Birth Defects Res. 109, 1672–1679. doi: 10.1002/bdr2.1178, PMID: 29251839 PMC5973814

[ref23] Hossian M. Mielke G. I. Nisar M. Tremblay M. S. Khan A. (2025). Global research on 24-hour movement behaviours guidelines in children and adolescents: a systematic review. Int. J. Behav. Nutr. Phys. Act. 22:108. doi: 10.1186/s12966-025-01809-5, PMID: 40781309 PMC12333077

[ref24] Huang T. Tarp J. Domazet S. L. Thorsen A. K. Froberg K. Andersen L. B.. (2015). Associations of adiposity and aerobic fitness with executive function and math performance in Danish adolescents. J. Pediatr. 167, 810–815. doi: 10.1016/j.jpeds.2015.07.009, PMID: 26256018

[ref25] Im S. Stavas J. Lee J. Mir Z. Hazlett-Stevens H. Caplovitz G. (2021). Does mindfulness-based intervention improve cognitive function? A meta-analysis of controlled studies. Clin. Psychol. Rev. 84:101972. doi: 10.1016/j.cpr.2021.101972 33582570

[ref26] Jordan L. C. Siciliano R. E. Cole D. A. Lee C. A. Patel N. J. Murphy L. K.. (2020). Cognitive training in children with hypoplastic left heart syndrome: a pilot randomized trial. Prog. Pediatr. Cardiol. 57:101185. doi: 10.1016/j.ppedcard.2019.101185 PMC12657031 41312086

[ref27] Kawaike Y. Nagata J. Furuya T. Koriyama C. Nakamura M. Sano A. (2019). Working memory-related prefrontal hemodynamic responses in university students: a correlation study of subjective well-being and lifestyle habits. Front. Behav. Neurosci. 13:213. doi: 10.3389/fnbeh.2019.00213, PMID: 31572144 PMC6754075

[ref28] Kjellenberg K. Ekblom Ö. Tarassova O. Fernström M. Nyberg G. Ekblom M. M.. (2024). Short, frequent physical activity breaks improve working memory while preserving cerebral blood flow in adolescents during prolonged sitting - AbbaH teen, a randomized crossover trial. BMC Public Health 24:2090. doi: 10.1186/s12889-024-19306-y, PMID: 39095724 PMC11295579

[ref29] Lambek R. Shevlin M. (2011). Working memory and response inhibition in children and adolescents: age and organization issues. Scand. J. Psychol. 52, 427–432. doi: 10.1111/j.1467-9450.2011.00899.x, PMID: 21722136

[ref30] Lenroot R. K. Giedd J. N. (2010). Sex differences in the adolescent brain. Brain Cogn. 72, 46–55. doi: 10.1016/j.bandc.2009.10.008, PMID: 19913969 PMC2818549

[ref31] Liu B. Shi P. Jin T. Feng X. (2025). Associations between meeting 24h movement behavior guidelines and cognition, gray matter volume, and academic performance in children and adolescents: a systematic review. Arch. Public Health 83:10. doi: 10.1186/s13690-024-01493-0, PMID: 39794834 PMC11720839

[ref32] Liu L. Xin X. Zhang Y. (2025). The effects of physical exercise on cognitive function in adolescents: a systematic review and meta-analysis. Front. Psychol. 16:1556721. doi: 10.3389/fpsyg.2025.1556721, PMID: 40792082 PMC12337486

[ref33] López-Vicente M. Garcia-Aymerich J. Torrent-Pallicer J. Forns J. Ibarluzea J. Lertxundi N.. (2017). Are early physical activity and sedentary behaviors related to working memory at 7 and 14 years of age? J. Pediatr. 188, 35–41.e1. doi: 10.1016/j.jpeds.2017.05.079, PMID: 28688631

[ref34] Ludyga S. Gerber M. Kamijo K. (2022). Exercise types and working memory components during development. Trends Cogn. Sci. 26, 191–203. doi: 10.1016/j.tics.2021.12.004, PMID: 35031211

[ref35] Morra S. Howard S. J. Loaiza V. M. (2025). Working memory and executive functions: theoretical advances. J. Cogn. 8:15. doi: 10.5334/joc.424, PMID: 39803173 PMC11720567

[ref36] Muppalla S. K. Vuppalapati S. Reddy Pulliahgaru A. Sreenivasulu H. (2023). Effects of excessive screen time on child development: an updated review and strategies for management. Cureus. 15:e40608. doi: 10.7759/cureus.40608, PMID: 37476119 PMC10353947

[ref37] National Health Commission of the People’s Republic of China (National Center for Disease Control and Prevention). (2024) Physical activity level evaluation for children and adolescents aged 7 - 18 years.WS/T 10008-2023, health industry standard, China. Available online at: https://www.ndcpa.gov.cn/doc/ucap/1625444428300685312/document/20231227/Jqf0KKhL.pdf

[ref38] Pesce C. Croce R. Ben-Soussan T. D. Vazou S. McCullick B. Tomporowski P. D.. (2016). Variability of practice as an interface between motor and cognitive development. Int. J. Sport Exerc. Psychol. 17, 133–152. doi: 10.1080/1612197X.2016.1223421

[ref39] Pickersgill J. W. Turco C. V. Ramdeo K. Rehsi R. S. Foglia S. D. Nelson A. J. (2022). The combined influences of exercise, diet and sleep on neuroplasticity. Front. Psychol. 13:831819. doi: 10.3389/fpsyg.2022.831819, PMID: 35558719 PMC9090458

[ref40] Ramer J. D. Santiago-Rodríguez M. E. Vukits A. J. Bustamante E. E. (2022). The convergent effects of primary school physical activity, sleep, and recreational screen time on cognition and academic performance in grade 9. Front. Hum. Neurosci. 16:1017598. doi: 10.3389/fnhum.2022.1017598, PMID: 36438639 PMC9687380

[ref41] Rosenberger M. E. Fulton J. E. Buman M. P. Troiano R. P. Grandner M. A. Buchner D. M.. (2019). The 24-hour activity cycle: a new paradigm for physical activity. Med. Sci. Sports Exerc. 51, 454–464. doi: 10.1249/MSS.0000000000001811, PMID: 30339658 PMC6377291

[ref42] Sampasa-Kanyinga H. Colman I. Goldfield G. S. Janssen I. Wang J. L. Podinic I.. (2020). Combinations of physical activity, sedentary time, and sleep duration and their associations with depressive symptoms and other mental health problems in children and adolescents: a systematic review. Int. J. Behav. Nutr. Phys. Act. 17:72. doi: 10.1186/s12966-020-00976-x, PMID: 32503638 PMC7273653

[ref43] Sanz-Martín D. Ubago-Jiménez J. L. Ruiz-Tendero G. Zurita-Ortega F. Melguizo-Ibáñez E. Puertas-Molero P. (2022). The relationships between physical activity, screen time and sleep time according to the adolescents’ sex and the day of the week. Healthcare (Basel) 10:1955. doi: 10.3390/healthcare10101955, PMID: 36292402 PMC9601728

[ref44] Schwaighofer M. Fischer F. Bühner M. (2015). Does working memory training transfer? A meta-analysis including training conditions as moderators. Educ. Psychol.-US. 50, 138–166. doi: 10.1080/00461520.2015.1036274

[ref45] Sewell K. R. Erickson K. I. Rainey-Smith S. R. Peiffer J. J. Sohrabi H. R. Brown B. M. (2021). Relationships between physical activity, sleep and cognitive function: a narrative review. Neurosci. Biobehav. Rev. 130, 369–378. doi: 10.1016/j.neubiorev.2021.09.003, PMID: 34506842

[ref46] Shi P. Tang Y. Zhang Z. Feng X. Li C. (2022). Effect of physical exercise in real-world settings on executive function of typical children and adolescents: a systematic review. Brain Sci. 12:1734. doi: 10.3390/brainsci12121734, PMID: 36552193 PMC9775424

[ref47] Su X. Hassan M. A. Kim H. Gao Z. (2024). Comparative effectiveness of lifestyle interventions on children’s body composition management: a systematic review and network meta-analysis. J. Sport Health Sci. 14:101008. doi: 10.1016/j.jshs.2024.101008 39510316 PMC11863321

[ref48] Tang Y. Y. Yang L. Leve L. D. Harold G. T. (2012). Improving executive function and its neurobiological mechanisms through a mindfulness-based intervention: advances within the field of developmental neuroscience. Child Dev. Perspect. 6, 361–366. doi: 10.1111/j.1750-8606.2012.00250.x, PMID: 25419230 PMC4238887

[ref49] Tapia-Serrano M. Á. López-Gil J. F. Sevil-Serrano J. García-Hermoso A. Sánchez-Miguel P. A. (2023). What is the role of adherence to 24-hour movement guidelines in relation to physical fitness components among adolescents? Scand. J. Med. Sci. Sports 33, 1373–1383. doi: 10.1111/sms.14357, PMID: 36951638

[ref50] Tapia-Serrano M. A. Sevil-Serrano J. Sánchez-Miguel P. A. López-Gil J. F. Tremblay M. S. García-Hermoso A. (2022). Prevalence of meeting 24-hour movement guidelines from pre-school to adolescence: a systematic review and meta-analysis including 387,437 participants and 23 countries. J. Sport Health Sci. 11, 427–437. doi: 10.1016/j.jshs.2022.01.005, PMID: 35066216 PMC9338333

[ref51] Tee J. Y. H. Gan W. Y. Tan K. A. Chin Y. S. (2018). Obesity and unhealthy lifestyle associated with poor executive function among Malaysian adolescents. PLoS One 13:e0195934. doi: 10.1371/journal.pone.0195934, PMID: 29664932 PMC5903659

[ref52] Tremblay M. S. Carson V. Chaput J. P. Connor Gorber S. Dinh T. Duggan M.. (2016). Canadian 24-hour movement guidelines for children and youth: an integration of physical activity, sedentary behaviour, and sleep. Appl. Physiol. Nutr. Metab. 41, S311–S327. doi: 10.1139/apnm-2016-0151 27306437

[ref53] Troiano R. P. Berrigan D. Dodd K. W. Mâsse L. C. Tilert T. McDowell M. (2008). Physical activity in the United States measured by accelerometer. Med. Sci. Sports Exerc. 40, 181–188. doi: 10.1249/mss.0b013e31815a51b3 18091006

[ref54] van Sluijs E. M. F. Ekelund U. Crochemore-Silva I. Guthold R. Ha A. Lubans D.. (2021). Physical activity behaviours in adolescence: current evidence and opportunities for intervention. Lancet 398, 429–442. doi: 10.1016/S0140-6736(21)01259-9, PMID: 34302767 PMC7612669

[ref55] Walsh J. J. Barnes J. D. Cameron J. D. Goldfield G. S. Chaput J. P. Gunnell K. E.. (2018). Associations between 24 hour movement behaviours and global cognition in US children: a cross-sectional observational study. Lancet Child Adolesc Health 2, 783–791. doi: 10.1016/S2352-4642(18)30278-5, PMID: 30268792 PMC6298223

[ref56] Wilhite K. Booker B. Huang B. H. Antczak D. Corbett L. Parker P.. (2023). Combinations of physical activity, sedentary behavior, and sleep duration and their associations with physical, psychological, and educational outcomes in children and adolescents: a systematic review. Am. J. Epidemiol. 192, 665–679. doi: 10.1093/aje/kwac212, PMID: 36516992 PMC10089066

[ref57] Wu H. T. Gan M. L. Yin X. J. Wu H. P. Shen J. B. Ma Y. Y.. (2023). Correlation between physical activity and depressive symptoms in adolescents. Chin. J. School Health 44, 672–676. doi: 10.16835/j.cnki.1000-9817.2023.05.008

[ref58] Zayed K. Jansen P. (2018). Gender differences and the relationship of motor, cognitive and academic achievement in Omani primary school-aged children. Front. Psychol. 9:2477. doi: 10.3389/fpsyg.2018.02477, PMID: 30618922 PMC6304386

[ref59] Zhu L. N. Yin H. C. Chen A. G.. (2020). Cortical thickness mediates the association between aerobic capacity and executive function in young adults. China Sport Sci. Technol. 56, 48–54. doi: 10.16470/j.csst.2020127

